# Optical Properties and Amplified Spontaneous Emission of Novel MDMO-PPV/C500 Hybrid

**DOI:** 10.3390/polym9020071

**Published:** 2017-02-17

**Authors:** Rasha A. Abumosa, Bandar Ali Al-Asbahi, Mohamad Saleh AlSalhi

**Affiliations:** 1 Department of Physics & Astronomy, College of Science, King Saud University, Riyadh 11451, Saudi Arabia; r.abumosa@hotmail.com; 2 Research Chair in Laser Diagnosis of Cancers, College of Science, King Saud University, Riyadh 11451, Saudi Arabia; 3 Department of Physics, Faculty of Science, Sana’a University, P.O. Box 12544 Sana’a, Yemen

**Keywords:** optical properties, quantum yield, amplified spontaneous emission, energy transfer

## Abstract

The influence of the solvent nature on optical properties of poly[2-methoxy-5-3,7-dimethyloctyloxy-1,4-phenylenevinylene] (MDMO-PPV)/Coumarine 500 (C500) have been investigated. In addition, the amplified spontaneous emission (ASE) from MDMO-PPV and efficient energy transfer between the MDMO-PPV and C500 has been verified. The MDMO-PPV was dissolved in aromatic and nonaromatic solvents, while the solution blending method was employed to prepare the MDMO-PPV:C500 hybrid. The quantum yield of the MDMO-PPV was found to increase with the reduction of a few factors such as polarity index of the solvent, absorption cross section (σ_a_), emission cross section (σ_e_), and extinction coefficient (ε_max_). The fluorescence spectra of the MDMO-PPV appears from two vibronic band transitions (0-0, 0-1) and the ASE occurs at 0-1 transition, which was verified by the ASE from MDMO-PPV. The MDMO-PPV in toluene exhibited the best ASE efficiency due to its high quantum yield compared with other solvents. Strong overlap between the absorption spectrum of MDMO-PPV and emission spectrum of C500 confirmed the efficient energy transfer between them. Moreover, the ASE for energy transfer of the MDMO-PPV:C500 hybrid was proved.

## 1. Introduction

Conducting polymers (CPs) have attracted great interest for their unique optical properties including having a variety of colors, tuning of the energy band gap with minor structural modification, fast switching ability, and high optical contrast in the near infra-red (NIR) and visible regions [1,2,3,4]. Similar to inorganic semiconductors, CPs are considered unique photonic materials that combine their mechanical and structural properties together with the optoelectric characteristics of materials [5,6]. During the last two decades, the application of CPs has been widely reported in several optoelectronic devices such as: sensors [7,8], solar cells [1,9], light emitting electrochemical cells [10,11], and light emitting diodes [12,13]. Moreover, conducting CPs have been utilized as lasers and gain media for optical amplifiers with tunable features in the visible range of the electromagnetic spectrum [14,15,16].

On the other hand, coumarine derivatives have recently attracted the attention of researchers due to their physiological activities and optical properties. Due to their high emission quantum yield and photostability, they are broadly used as laser dyes. Moreover, the chemical variation of laser dyes leads to the modification of emission wavelengths continuously throughout the visible spectrum. Generally, the first material systems used to investigate the laser action and other optical processes in microcavities are liquid dye lasers. In liquid dye lasers, the laser wavelength tuning is achieved either by varying the optical path length in the cavity or by changing the dye concentration. The solvent tuning method can be used to change the optical path of the cavity, where the refractive index in the cavity can be changed by changing the solvent of the dye. Consequently, the laser wavelength may be changed due to the interaction between solvent molecules and dye molecules.

In fact, the use of two materials with complementary *p*-type (donor) and *n*-type (acceptor) electronic properties is crucial to control the light emission which has a significant application in the area of characterizing laser materials. Mainly, the excitation of the donor can result in a non-radiative energy transfer to the acceptor, and then enhancement of an acceptor emission and a decrease in a donor emission. This fact is related to two main conditions. The first condition is the fluorophores of an acceptor and a donor must be very close together (less than 10 nm). The second is that the acceptor excitation is of similar energy as that of the donor emission. The enhancement of an acceptor emission with the reduction of a donor emission has led to fluorescence resonance energy transfer (FRET) being referred to as a ‘spectroscopic ruler’ [17]. In comparison to the pristine host system, the use of highly efficient acceptor materials leads to an enhancement of the quantum efficiency of the system [18,19]. In the context of lasers, efficient energy transfer between several donor–acceptor pairs has been reported to enhance the laser performance and to extend the spectral range of laser operation [20,21]. In donor–acceptor pairs, self-absorption is significantly reduced because the emission region is shifted away from absorption. Also, concentration quenching is reduced where the concentration of the acceptor (1%–10%) is negligible. The threshold of amplified spontaneous emission (ASE) can be reduced by both of these factors [18].

The energy transfer mechanism principally requires a spectral overlap between the acceptor absorption and the donor emission. The rate of excitation energy transfer also depends on a few parameters, namely the concentration of the acceptor molecule, the emission quantum yield of the donor, the light absorption ability of the acceptor, and the relative orientation of the acceptor and donor transition dipoles.

Although many reports have been published on ASE and energy transfer in the mixtures of laser dyes/CPs, no report has been written on the incorporation of poly[2-methoxy-5-3,7-dimethyloctyloxy-1,4-phenylenevinylene] (MDMO-PPV) into Coumarine 500 (C500) which dealt with the ASE and energy transfer. In the current paper, we report the optical properties of MDMO-PPV. Moreover, the observation of ASE and energy transfer in the mixtures of (C500) as donor with MDMO-PPV as acceptor in aromatic and nonaromatic solvents under pulsed optical excitation will be proven in detail.

## 2. Experimental Procedures

### 2.1. Materials

Conjugated (MDMO-PPV) polymer was purchased from Sigma-Aldrich Corporation (St. Louis, MO, USA), whereas C500 laser dye was purchased from Exciton (Dayton, OH, USA). It was dissolved in common aromatic solvents, such as toluene and chlorobenzene (CB), and in nonaromatic solvents, namely chloroform and tetrahydrofuran (THF). All solvents were produced by Fluka (Buchs, Switzerland) with a purity of 99.8% for each solvent.

### 2.2. Characterization

The absorption spectra were recorded using a UV-visible spectrophotometer (UV-1650 PC; Shimadzu, Kyoto, Japan) with a slit of 2 nm and scan speed of 200 nm/min. The behavior of the molecules in the excited state was investigated by fluorescence spectra, which was obtained by Spectrofluorometer (RF-5301PC; Shimadzu). The fluorescence spectra were recorded with an excitation wavelength of 480 nm, excitation and emission slit of 5 nm, scan speed medium, and high sensitivity.

The ASE was obtained under high source Q-switched (neodymium-doped yttrium aluminium garnet; Nd:Y_3_Al_5_O_12_) Nd:YAG laser (532 nm, Brilliant B of Quantel, Les Ulis, France). The pumping energy was taken from 6 to 12 mJ with a pulsed width of 11 ns and the reputation rate kept on 10 pulses per second. The laser beam was focused onto the sample using a cylindrical lens of focal length 5 cm. The ASE was collected from the edge of the side of the quartz cuvette (1 cm ×1 cm × 4 cm) using a 10 cm focal length lens and detected by a 1 mm entrance slit of a M266 camera (solar LS, Stebenev lane, Minsk, Belarus).

The energy transfer properties of the conjugated polymer/dye hybrid was investigated using the UV-visible spectrophotometer, spectrofluorometer with excitation wavelength of 388 nm and a UV laser beam (355 nm) of 10 Hz, Q-switched Nd:YAG laser. The donor and acceptor were kept at fixed a concentration at 2 and 0.67 mg/mL, respectively. Four different volume ratios of the donor:acceptor, namely, 50:30, 50:50, 50:90, and 50:100 were prepared to study the ASE for energy transfer.

## 3. Results and Discussion

### 3.1. Optical Properties of the MDMO-PPV

**Figure 1:**
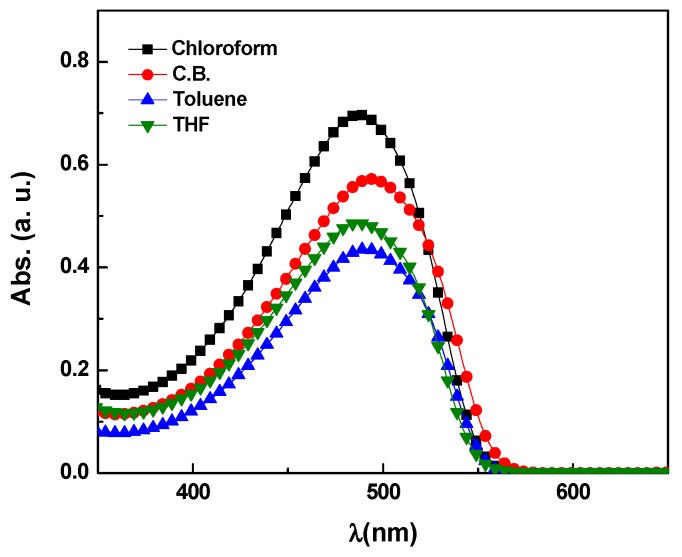
Optical absorbance spectra of 0.01 mg/mL of poly[2-methoxy-5-3,7-dimethyloctyloxy-1,4-phenylenevinylene] (MDMO-PPV) in different solvents.

Figure 1 illustrates the absorption spectra of MDMO-PPV at fixed a concentration of 10^−2^ mg/mL in various solvents. It can be noted that the absorption peak shifted slightly with variation in the type of the solvent. The absorption peak of MDMO-PPV in the aromatic solvents, which have the lowest polarity as mentioned in Table 1, is around 493 nm whereas it is at 486 and 488 nm for chloroform and THF, respectively. This observation can be attributed to the change in the polarity of the solvent, where the absorption peak slightly red shift with decreasing the solvent polarity.

**Figure 2:**
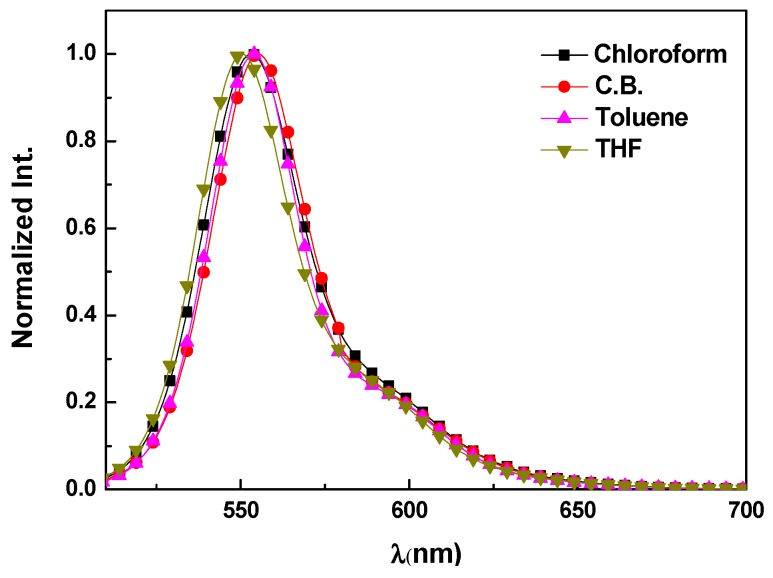
Fluorescence spectra of 0.01 mg/mL of MDMO-PPV in different solvents.

The solvent effect on the fluorescence spectra of MDMO-PPV (10^−2^ mg/mL) was investigated with different solvents, as shown in Figure 2. Two peaks are observed due to 0-0 and 0-1 transitions. The 0-0 transition is located at 555, 554, 553, and 550 nm for CB, toluene, chloroform, and THF, respectively. The 0-1 transition appeared close to 585 nm, which can be attributed to the formation of aggregated species where there fluorescence is roughly the same as the vibronic transition of the isolated polymer chains. It is clearly observed that the aggregation of MDMO-PPV molecules happens more readily in aromatic solvents such as toluene which interacts preferentially with the aromatic backbone of the polymer chain. The nonaromatic solvents such as THF are more likely to interact with the side groups of the polymer, thus, causing the polymer chains to form tight coils and minimize the exposure of the backbone and reduce the formation of aggregates [22]. Consequently, fluorescence spectra are slightly blue shifted in THF relative to other solutions, where the degree of the aggregation is hindered in THF solvent and promoted in toluene solution. This finding is in good agreement with previous studies on another conjugated polymer from the same PPV derivatives, namely poly[2-methoxy-5-(2-ethylhexyloxy)-1,4phenylenevinylene] (MEH-PPV) [23,24].

The fluorescence quantum yield ($\emptyset_{f}$), was calculated for MDMO-PPV solutions at a concentration of 10^−2^ mg/mL by using the following equation [25].

(1) $$\emptyset_{f}\left(S\right)=\;\emptyset_{f}\left(R\right)\frac{\int I_{S}\left(\bar{\nu }\right)d\left(\bar{\nu }\right)}{\int I_{R}\left(\bar{\nu }\right)d\left(\bar{\nu }\right)}\;\cdot {\left(\frac{n_{S}}{n_{R}}\right)}^{2}\;\left(\frac{1-\;10^{-A_{R}}}{1-\;10^{-A_{S}}}\right)\;$$

where the indices *R* and *S* refer to reference and sample, respectively. The integrals signify the corrected fluorescence peak area at the excitation wavelength (in this study at 488 nm); *n* is the refractive index of the solvent while *A* is the absorbance. The reference fluorophore was Rhodamine 6G dissolved in methanol, which has a standard $\emptyset_{f}\left(R\right)$ value of 0.94 [26]. Regarding to the results in Table 1, it can be concluded that the effect of the solvents’ nature on the emission spectra is complex. The complexity can be ascribed to several factors besides solvent polarity [27]. Table 1 shows the strong dependence of $\emptyset_{f}\;$on the solvent nature as well as other spectral properties such as absorption cross section (σ_a_), emission cross section (σ_e_), and extinction coefficient (ε_max_). It is not easy to determine which factor is prevalent in a particular experimental system, and typically more than one factor will concurrently affect the fluorophore. Nevertheless, solvent polarity is usually the essential factor to be mentioned when considering solvent effect [25]. Therefore, from Table 1, the highest value of $\emptyset_{f}$ noted in toluene compared with that of other solvents is not only the result of its lowest polarity but also the result of its lowest σ_a_, σ_e_, and ε_max_ as well. In other words, the lowest value of $\emptyset_{f}$ in chloroform is the combined result of its high polarity, σ_a_, σ_e_, and ε_max_.

### 3.2. Amplified Spontaneous Emission and Energy Transfer

**Figure 3:**
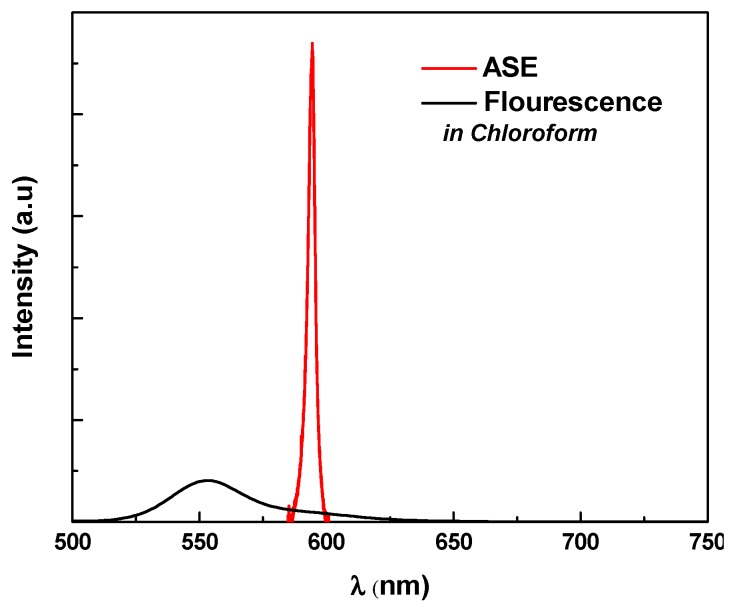
Comparison of the fluorescence spectra of the MDMO-PPV to the amplified spontaneous emission (ASE).

To show the amplified spontaneous emission (ASE) from the MDMO-PPV, the ASE peak must be observed at the range of its fluorescence spectra. For this purpose, the fluorescence and ASE spectra of the MDMO-PPV dissolved in chloroform, as an example, are illustrated in Figure 3. It can be clearly seen that the fluorescence spectrum has two peaks, one at 553 nm and the other close to 585 nm, and the ASE peak is located at 585 nm with narrowed 3.3 nm. This finding means that the fluorescence appears from two vibronic band transitions (0-0, 0-1) and the ASE occurs at the 0-1 transition, which is shown in the ASE from the MDMO-PPV. The existence of the ASE at the 0-1 transition rather than the 0-0 transition may be explained by a higher net gain of the 0-1 peak than that of the 0-0 peak because it is farther from the absorption edge and, therefore, has less reabsorption loss [28,29].

**Figure 4:**
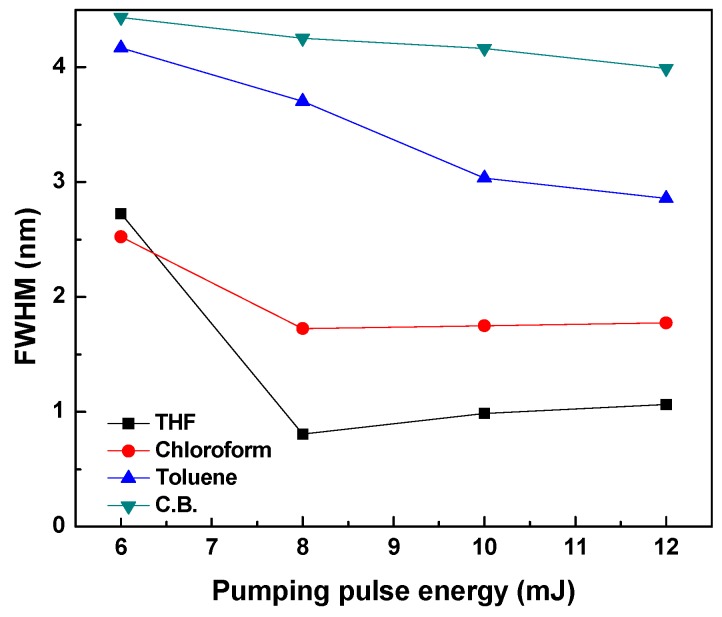
ASE line width versus pulse energy for MDMO-PPV solutions with different solvents at a constant concentration (0.67 mg/mL).

The ASE full width at half maximum (FWHM) of MDMO-PPV dissolved in various solvents at a specific concentration of 0.67 mg/mL is demonstrated in Figure 4. It can be observed that the FWHM of the ASE peak is decreases with increasing pumping energy in all solvents. The narrowest FWHM was for nonaromatic solvents (THF and chloroform), which have the highest polarity compared with that of other aromatic solvents. Once the pumping energy exceeded 8 mJ, the ASE narrowing of the MDMO-PPV in THF and chloroform did not change and subsequently gain narrowing becomes constant. These results stem from the fact that the solvent nature is playing a significant role in producing ASE [29].

**Figure 5:**
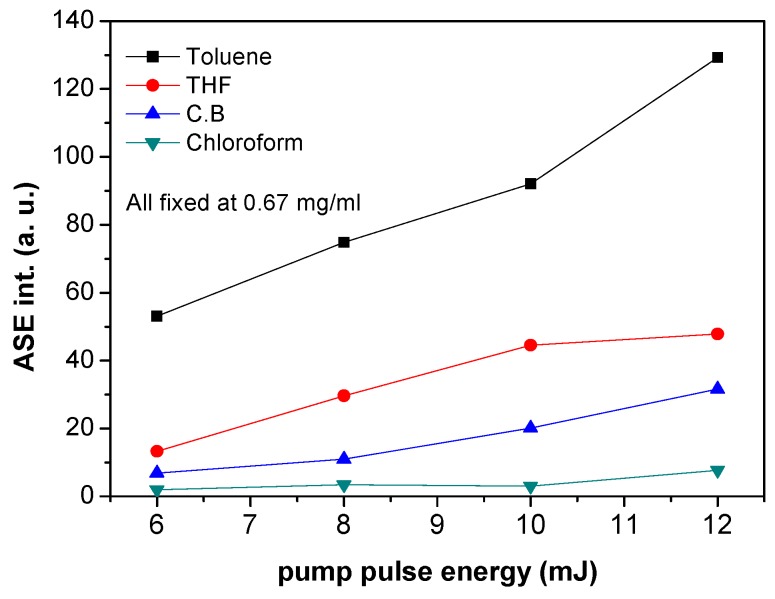
ASE intensity of MDMO-PPV dissolved in different solvents for different pumping energy and a fixed concentration at 0.67 mg/mL.

Figure 5 illustrates the dependence of ASE intensity on the pumping energy for various solvents at a fixed concentration of 0.67 mg/mL. The ASE intensity increases with increasing pumping energy. This is due to the optical scattering by the MDMO-PPV monomers which increase the path length of the emitted light inside the gain region. When the photon travels in the gain region, it may induce the stimulated emission of a second photon as the pump power increases, and, thus, the gain length is reduced. Eventually the gain length at frequencies near the maximum of the gain spectrum approaches the average path length of the photons in the gain region. Consequently, the probability of a photon being generated by a second photon before leaving the gain region increases [30]. Moreover, it is clear that MDMO-PPV dissolved in toluene exhibited the highest ASE intensity compared with that of the other solvents, whereas the lowest intensity was for MDMO-PPV dissolved in chloroform. This is may be due to the fact that the quantum yield of MDMO-PPV dissolved in toluene is the greatest compared with that of the other solvents, while the quantum yield of MDMO-PPV dissolved in chloroform is the lowest.

**Figure 6:**
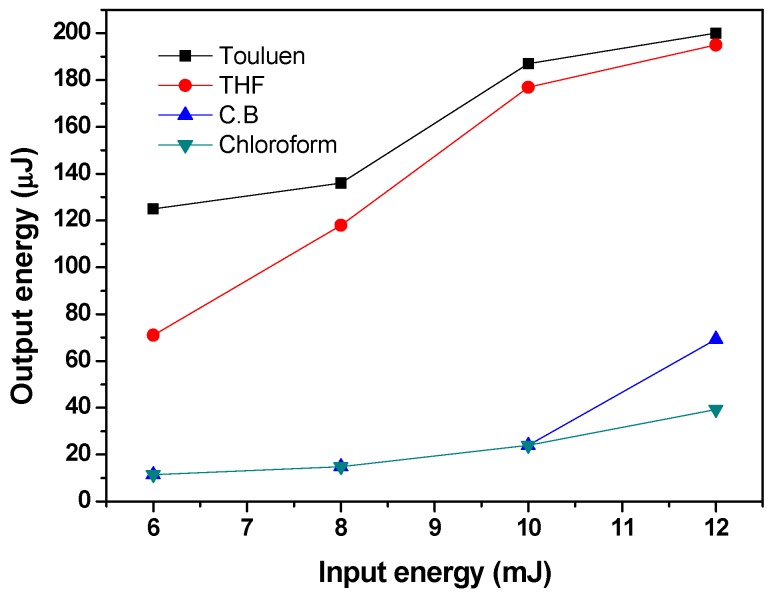
Input vis. Output energy relationship of MDMO-PPV dissolved in different solvents at a fixed concentration of 0.67 mg/mL.

Figure 6 shows the relationship between output and input energy for different MDMO-PPV dissolved in various solvents with a fixed concentration at 0.67 mg/mL. The ordinary linear dependence of the output pulse energy of the polymer solution as a function of the input pulse energy of the Nd:YAG laser can be seen. Moreover, the nature of the solvents has different effects on the Output energy. Toluene is the best solvent which gives the highest ASE efficiency, whereas chloroform is the worst solvent compared with the other solvents. This finding can be ascribed to the highest quantum yield for MDMO-PPV dissolved in toluene and lowest yield in chloroform, compared with other solvents. On other hand, the highest ASE efficiency is due to the fact that toluene has the lowest polarity index, whereas the lowest ASE efficiency in chloroform is due to its high polarity index compared with that of other solvents.

**Figure 7:**
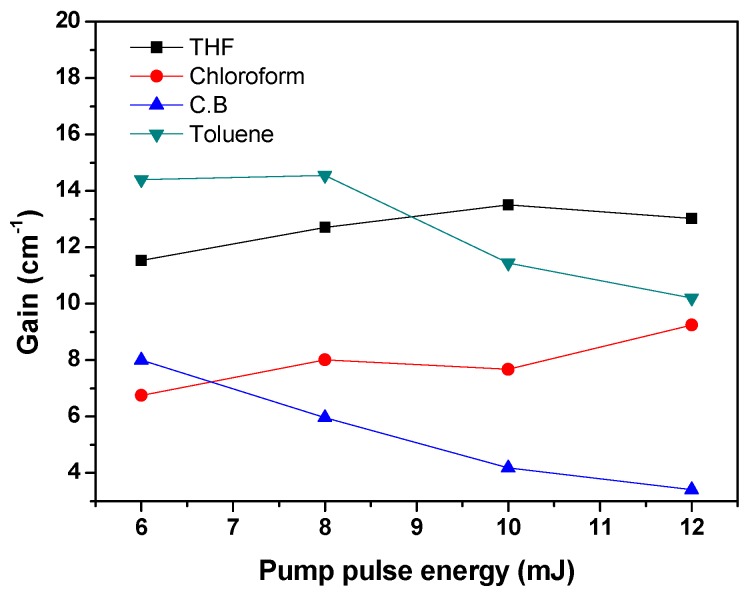
Optical gain of MDMO-PPV dissolved in different solvents versus pump pulse energy.

Figure 7 shows the dependence of the ASE gain of MDMO-PPV dissolved in various solvents on pumping energy at a fixed concentration of 0.67 mg/mL. The figure indicates that the optical gain decreases by increasing the pumping energy for MDMO-PPV dissolved in aromatic solvents (Toluene and CB), while it increases by increasing the pumping energy for MDMO-PPV dissolved in nonaromatic solvents (THF and chloroform) which possess a high polarity index compared with that of the aromatic solvents.

**Figure 8:**
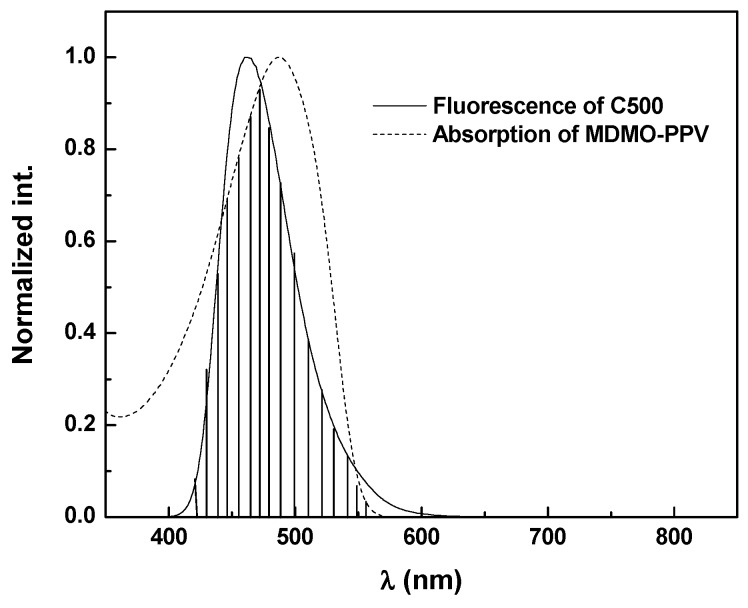
Overlap of the absorption spectra of MDMO-PPV with the emission spectra of Coumarine 500 (C500) in chloroform.

The verification of non-radiative energy transfer conditions between MDMO-PPV as the acceptor and C500 as donor was shown in Figure 8. For this purpose, the MDMO-PPV and C500solutions were prepared in chloroform at a concentration of 10^−2^ mg/mL and 10^−3^ mg/mL, respectively. The strong overlap between absorption spectrum of MDMO-PPV and emission spectrum of C500 can be clearly observed. This overlap shows that non-radiative energy transfer from C500 to MDMO-PPV is clearly possible.

**Figure 9:**
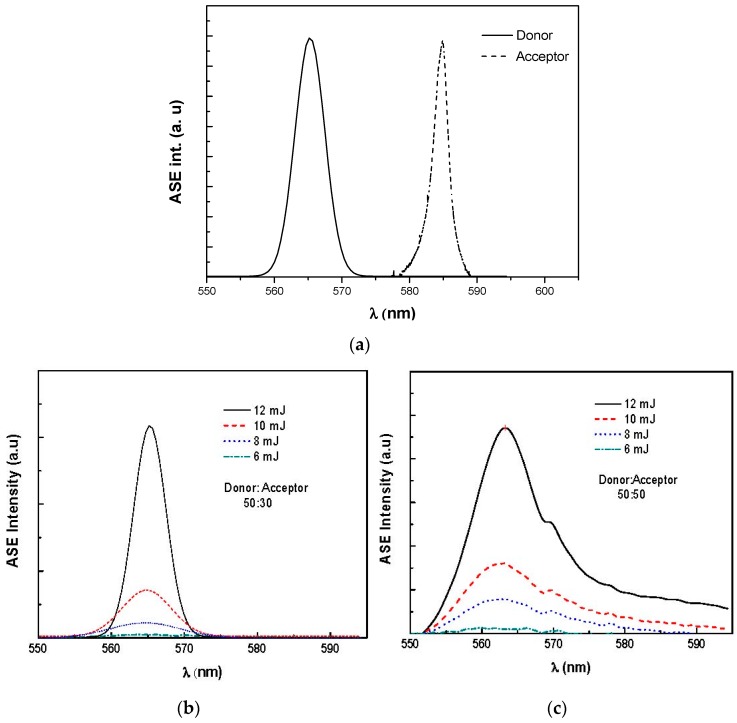

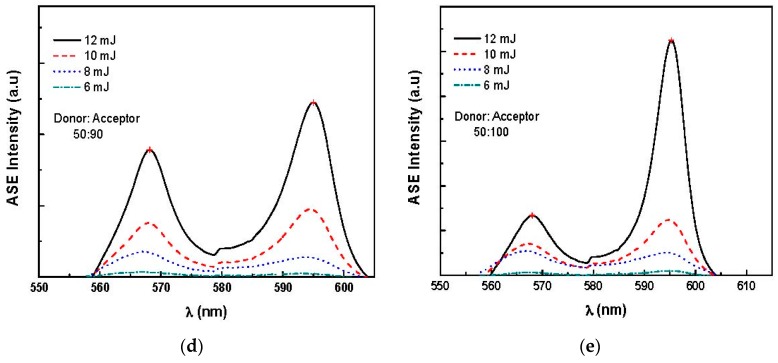
Energy transfer from C500 to MDMO-PPV at different ratios for different pumping energies in chloroform. (**a**) pure donor and pure acceptor, (**b**) 50:30, (**c**) 50:50, (**d**) 50:90, and (**e**) 50:100.

The ASE for energy transfer between the donor and acceptor hybrid was studied under high source Nd:YAG laser excitation (355 nm) and various pump pulse energies ranging from 6 to 12 mJ. As shown in Figure 9, an ASE from C500 occurred at 565 nm in absence of the MDMO-PPV. Similarly, without the donor, there is only one ASE band at 585 nm due to the 0-1 vibronic transition. Both of these findings of the ASE wavelengths are independent on pump power or concentration of either donor or acceptor. However, in the presence of both donor and acceptor with a volume ratio up to 50:50 under various pump powers, the ASE from acceptor disappeared while the ASE is almost totally from the donor at around 565 nm. When the volume ratio exceeded 50:50, the peak of the acceptor appeared with a dramatic decrease in the donor ASE intensity. This means that some of the excitation energy was emitted and the rest was sapped by the acceptor, which is indicative of an extraordinarily efficient energy transfer process from molecules of the C500 to monomers of the MDMO-PPV. Moreover, at higher concentrations of acceptor, i.e., 50:90, two ASE bands appear. The first one is around 565 nm, due to the donor, and the second is around 595 nm, related to the 0-1 vibronic transition of the acceptor. There are two reasons for the red shifting of the ASE peak (from 585 to 595 nm) at the higher content of the acceptor: The first is the absorption of the MDMO-PPV overlaps the short-wavelength side of its fluorescence spectrum. The second is the competition between the stimulated emission mechanism and the energy transfer from the donor to the acceptor [18,31]. Once the volume ratio exceeded 50:90, the ASE peak at 595 nm, which related to the acceptor, became dominant; while the peak of the donor at 565 nm was quenched dramatically. This observation signified that the energy transfer from the C500 molecules to the considerable number of MDMO-PPV monomers was not emitted as fluorescence but converted into heat, and this implies that some of the monomers acted as dark quenchers without any fluorescence [32]. This finding is consistent with previous reports where the ASE was found from the donor for acceptor concentration below 10%, whereas above this ratio, the ASE was from the acceptor [18,31].

## 4. Conclusions

The optical properties and ASE of the MDMO-PPV were investigated in detail. The ASE from the donor/acceptor blend was successfully obtained. The results demonstrated that the fluorescence quantum yield was the greatest in toluene, whereas the lowest value was in chloroform. Furthermore, the solvent nature played a significant role in producing ASE as well. MDMO-PPV dissolved in toluene exhibited the highest ASE efficiency due to its high quantum yield compared with other solvents. The strong overlap between the absorption spectrum of MDMO-PPV and the emission spectrum of C500 confirmed that non-radiative energy transfer from C500 to MDMO-PPV was clearly possible. The ASE wavelength of the donor and acceptor are independent on pump power and concentration of either donor or acceptor. However, the ASE wavelength from a donor/acceptor hybrid is dependent on the volume ratio of donor to acceptor. When the volume ratio exceeded 50:90, the peak of the donor (565 nm) dramatically vanished where some of the monomers acted as dark quenchers without any fluorescence.

## Figures and Tables

**Table 1:** Spectral properties of conjugated MDMO-PPV polymer in different solvents at 10^−2^ mg/mL.

Solvent	Quantum yield %	Polarity index	ε_max_ × 10^7^ (M·cm)^−1^	σ_a_ × 10^−14^ (cm^2^)	σ_e_ × 10^−16^ (cm^2^)
Toluene	40.36	2.3	1.36	0.20	1.83
CB	33.02	2.7	2.23	0.26	2.87
THF	33.63	4.2	2.96	0.22	2.64
Chloroform	25.55	4.4	3.96	0.32	4.23
